# Phylogeny and evolution of *Lasiopodomys* in subfamily Arvivolinae based on mitochondrial genomics

**DOI:** 10.7717/peerj.10850

**Published:** 2021-03-16

**Authors:** Luye Shi, Likuan Liu, Xiujuan Li, Yue Wu, Xiangyu Tian, Yuhua Shi, Zhenlong Wang

**Affiliations:** 1 School of Life Sciences, Zhengzhou University, Zhengzhou, Henan, China; 2 School of Life Sciences, Qinghai Normal University, Xining, Qinghai, China

**Keywords:** Lasiopodomys, Mitochondrial genomes, Phylogenetic analysis, Arvivolinae

## Abstract

The species of *Lasiopodomys* Lataste 1887 with their related genera remains undetermined owing to inconsistent morphological characteristics and molecular phylogeny. To investigate the phylogenetic relationship and speciation among species of the genus *Lasiopodomys*, we sequenced and annotated the whole mitochondrial genomes of three individual species, namely *Lasiopodomys brandtii* Radde 1861,* L. mandarinus* Milne-Edwards 1871, and *Neodon* (*Lasiopodomys*) *fuscus* Büchner 1889. The nucleotide sequences of the circular mitogenomes were identical for each individual species of *L. brandtii*, *L. mandarinus*, and *N. fuscus*. Each species contained 13 protein-coding genes (PCGs), 22 transfer RNAs, and 2 ribosomal RNAs, with mitochondrial genome lengths of 16,557 bp, 16,562 bp, and 16,324 bp, respectively. The mitogenomes and PCGs showed positive AT skew and negative GC skew. Mitogenomic phylogenetic analyses suggested that *L. brandtii*, *L. mandarinus*, and *L. gregalis* Pallas 1779 belong to the genus *Lasiopodomys*, whereas *N. fuscus* belongs to the genus *Neodon* grouped with *N. irene*. *Lasiopodomys* showed the closest relationship with *Microtus fortis* Büchner 1889 and *M. kikuchii* Kuroda 1920, which are considered as the paraphyletic species of genera Microtus. *T_MRCA_* and niche model analysis revealed that *Lasiopodomys* may have first appeared during the early Pleistocene epoch. Further, *L. gregalis* separated from others over 1.53 million years ago (Ma) and then diverged into* L. brandtii* and *L. mandarinus* 0.76 Ma. The relative contribution of climatic fluctuations to speciation and selection in this group requires further research.

## Introduction

Although taxonomical and molecular systematics have led to some progress in the relationship between the genus *Lasiopodomys* and its related genera, numerous uncertainties remain unelucidated. The species belonging to this genus was first described by Lataste in 1887 as part of the Arvivolinae Gray 1821 (Cricetidae Fischer 1817) subfamily, which includes the genera *Phaiomys* Blyth 1863, *Microtus* Schrank 1798, and *Neodon* Horsfield 1841 (Allen, 1940; Corbet, 1978; Gromov & Polyakov, 1978; Liu et al., 2013; Wang, 2003). The genus *Lasiopodomys* includes three species from different colonial habitats of life—subterranean (*L. mandarinus* Milne-Edwards 1871), aboveground (*L. brandtii* Radde 1861), and plateau (*L. fuscus* Büchner 1889) by Wilson & Reeder (2005)—with relatively short tail and densely furred plantar surfaces. However, their generic taxonomy is not universally accepted, specifically in relation to *Phaiomys*, *Microtus*, and *Neodon*. Molecular data have revealed that the narrow-headed vole *Microtus gregalis* Pallas 1779 (formerly included in subgenus *Stenocranius* Katschenko 1901) is closely related to the species belonging to the genus *Lasiopodomys* (Abramson & Lissovsky, 2012). Morphological characteristics, such as karyotype (Gladkikh et al., 2016) and mating behavior (Zorenko & Atanasov, 2017), supported its current taxonomic status as *L. gregalis*. On the other hand, *L. fuscus* is nested in the genus *Neodon* Hodgson 1849 clade based on the longer length of ear and tail and greater number of inner angles in M_1_ and M^3^ compared with the genus *Lasiopodomys* (Liu et al., 2012a); moreover, *CLOCK*, *BMA1*, and *Cytb* gene sequences and their complete mitochondrial genomes supported this taxonomical status (Abramson et al., 2009a; Bannikova et al., 2010; Li, Lu & Wang, 2016a; Li et al., 2019; Liu et al., 2017). Recent studies have typically recognized *Lasiopodomys* as a separate genus that includes the species *L. mandarinus* and *L. brandtii*; *L. gregalis* was not widely accepted, whereas *L. fuscus* has been transferred to the genus *Neodon* and named *Neodon fuscus*.

According to fossils and molecular data, the genus *Lasiopodomys* originated and speciated during the Pleistocene epoch (∼2.58–0.012 million years ago (Ma)) when quaternary glaciations occurred in this period. Nuclear and mitochondrial phylogenetic estimates have shown that *Lasiopodomys* originated ∼2.4 Ma, whereas the division between *L. gregalis* and *Lasiopodomys* has been estimated to have occurred 1.8 Ma and that between *L. mandarinus* and *L. brandtii* was estimated at 0.5–0.95 Ma (Abramson et al., 2009b; Petrova et al., 2015; Li et al., 2017). However, chromosome analysis has shown that karyotype evolution has occurred between *L. mandarinus* and *L. brandtii* at ∼2.4 Ma, between *Lasiopodomys* and *L. gregalis* at 2.4 Ma, and between other *Microtus* species at 3 Ma (Gladkikh et al., 2016).

The species in the genus *Lasiopodomys* inhabit subterranean and aboveground environments and have recently become model species for comparative hypoxia adaptation (Dong et al., 2018; Sun et al., 2018). Species’ adaptation to low oxygen has been reported in numerous studies (Childress & Seibel, 1998; Dong et al., 2018; Nevo, 2013; Witt & Huerta-Sánchez, 2019), and most research has focused on animal models in an artificial environment or has compared them with subterranean rats to reveal the mechanisms of hypoxia (Ashur-Fabian et al., 2004; Malik et al., 2012; Malik et al., 2016). The differences in the environmental adaptability of proximal species are closely related to the historical events experienced during evolution, which play a key role in our understanding of the causes of current differences in life history among these species. However, the historical event that caused the *Lasiopodomys* species to adapt to a different environment has rarely been mentioned (Dong et al., 2018; Dong, Wang & Jiang, 2020).

Mitochondrial DNA are widely used to study the molecular ecology of animals because it is convenient and economical (Ballard & Rand, 2005; de Freitas et al., 2018; Kenechukwu, Li & An, 2018; Zhang et al., 2018). However, several studies have reported the limitations of mitochondrial DNA use (Galtier et al., 2009), such as recurrent horizontal transfer (Bergthorsson et al., 2003) and adaptive evolution (Bazin, Glémin & Galtier, 2006). The mitochondrial genome is involved in respiratory functions, which are closely associated with oxygen availability (Jain et al., 2016; Santore et al., 2002; Solaini et al., 2010).

In the present study, we sequenced the whole mitochondrial genomes of *L. mandarinus*, *L. brandtii*, and *N. fuscus*, which are species with three repeat individuals, using high-throughput sequencing technology and used the complete mitochondrial genomes of related species from the National Center for Biotechnology Information database to clarify the generic taxonomy of *Lasiopodomys* and evolutionary history of adaptation on aboveground and subsurface life. The findings of this research provide evolutionary information regarding the hypoxia adaptation of *Lasiopodomys*.

## Materials and Methods

### Material preparation and DNA sequencing

Total genomic DNA were extracted from the specimens of *L. mandarinus* (collected from 34°52′N, 113°85′E; Specimen No. LM023), *L. brandtii* (collected from 40°53′N, 116°38′E; Specimen No. LB003), and *N. fuscus* (collected from 34°9′N, 100°2′E; Specimen No. LF010) using the TIANamp Genomic DNA Extraction Kit (TIANGEN, DP304). All specimens were stored at the Animal Museum of Zhengzhou University. The Illumina NovaSeq 6000 (Illumina, San Diego, CA, USA) platform was used for sequencing the samples with a short-insert of 150 bp at ORI-GENE Company, Beijing (https://www.origene.com/).

### Genome assembly and annotation

NOVOPlasty 3.6 was used for *de novo* assembly using the mitochondrial genome of *L. mandarinus* (GenBank no. JX014233) as a reference (Dierckxsens, Mardulyn & Smits, 2017). All mitochondrial genomes were annotated using GeSeq (Tillich et al., 2017), OGDRAW (Lohse et al., 2013), and GB2sequin (Lehwark & Greiner, 2019) in the MPI-MP CHLOROBOX integrated web tool (https://www.mpimp-golm.mpg.de/chlorobox), which contains the function of the HMMER package for protein-coding genes (PCGs) and ribosomal RNA (rRNA) (Finn, Clements & Edd, 2011), and tRNAscan-SE v2.0.3 for transfer RNAs (tRNAs) (Lowe & Eddy, 1997). Adenine–thymine (AT) skew was calculated as AT skew = (A − T) / (A + T), whereas guanine–cytosine (GC) skew was calculated as GC skew = (G − C)/(G + C). Circular maps were drawn using the CGView Server V 1.0 web tool (http://stothard.afns.ualberta.ca/cgview_server/) for *L. mandarinus*, *L. brandtii*, *L. gregalis* (GenBank no. MN199169), and *N. fuscus* (Grant & Stothard, 2008).

### Molecular phylogenetic analysis and divergence time estimation

Phylogenetic analyses were performed on the whole mitochondrial genome sequences (Appendix S1). Besides the nine mitochondrial genomes that were acquired for the present study, five previously published mitochondrial genomes from *L. mandarinus*, *L. gregalis*, and *N*. *fuscus* were included; therefore, overall, 37 complete mitochondrial genome sequences from 23 species from the subfamily Arvivolinae were considered for phylogenetic analysis. Moreover, three species from *Cricetulus* Milne-Edwards 1867 were chosen as the outgroup. All these sequences were aligned using MAFFT v7.450 (Katoh & Standley, 2013). The nucleotide diversity of the PCGs of *Lasiopodomys* and Arvivolinae was determined using the DNASP v6.12.03 software (Rozas et al., 2017), and the best nucleotide substitution models were constructed using jMODELTEST 2.1.7 and selected using the Akaike information criterion (Darriba et al., 2012).

The phylogenetic relationships of the two different matrices as well as the whole mitochondrial genomes and PCG sequence matrices were constructed using the maximum likelihood (ML) approach in IQ-TREE v1.6.12 (Nguyen et al., 2015) and Bayesian analysis (BI) in the BEAST v1.8.4 program (Drummond & Rambaut, 2007). We conducted analysis using 5000 ultrafast bootstrap replicates and the best-fit model in the IQ-TREE software. To determine the maximum clade credibility trees of two different matrices, BEAST analyses were performed using the GTR+G+I substitution models identified above and the uncorrelated relaxed clocks for clock type (Drummond et al., 2006), Yule process for tree prior (Gernhard, 2008), and other default parameters. Each Markov chain Monte Carlo of 20,000,000 generations was sampled in every 10,000 generations. The effective sample sizes were estimated using Tracer v1.7 for all parameters more than 200 (Rambaut et al., 2018). Maximum clade credibility trees were constructed using TreeAnnotator v1.8.4 with a burn-in of the first 20% of the sampled trees (Drummond & Rambaut, 2007). Positive selection in all 13 PCGs was determined using branch models and branch-site models via phylogenetic analysis using ML (PAML4.7) programs (Yang, 2007). Branch models were used with the one-ratio model, i.e., all the species had the same *ω* ratio, and the *ω* = 1 model, with all species in natural selection. Based on the phylogenetic tree, we estimated the *ω* values of each PCG. The branch-site models used all *Lasiopodomys* species as the foreground branches, and the likelihood ratio test (LRT) was conducted to assess the statistical significance of positive selection.

The molecular divergence time was estimated using the Yule and birth–death processes for trees before implementing phylogeny construction using BEAST v1.8.4 (Gernhard, 2008; Heath, Huelsenbeck & Stadler, 2014). Marginal likelihood estimation for path sampling and stepping-stone sampling (Xie et al., 2011) using 5,000,000 in chain lengths of 500 path steps was used to sample the likelihood of every 5,000 chains (Baele et al., 2012; Baele et al., 2013). We applied three constraints to calibrate the tree at three prior nodes: (1) the divergence time of the Taiwan vole, *Microtus kikuchii* Kuroda 1920, and the reed vole *Microtus fortis*, of which the split between the subgenus *Alexandromys* Ognev 1914 and *Pallasiimus* Schrank 1798 was estimated via molecular clock analysis at ∼1.19 ± 0.19 Ma (Bannikova et al., 2010; Gao et al., 2017), (2) the earliest known fossil of *Eothenomys* Allen 1924 at 2.0 Ma (Liu et al., 2012a; Kohli et al., 2014), and (3) the oldest fossil of Arvicola, which was estimated at 3.0–3.5 Ma (Abramson et al., 2009a; Chen et al., 2012); we used the mean value of 3.25 Ma.

### Ecological niche modeling

The maximum entropy (Maxent) method was used to predict the current potential geographic distributions of *L. mandarinus*, *L. brandtii*, *L. gregalis*, and *N. fuscus* as well as their suitable distributions during the mid-Holocene, 6,000 years ago (kya), Last Glacial Maximum (LGM; 22 kya), and Last Interglacial (LIG; 120–140 kya) epochs (Phillips, Anderson & Schapire, 2006; Elith et al., 2011). Presence records were obtained for all four species according to the GBIF database and published papers (Appendix S2). Climatic variables with 19 bioclimatic layers were obtained from the database WorldClim version 1.4 at a resolution of 2.5 arc-minute grid format (Hijmans et al., 2005). The potential distributions of the species during the LGM and Holocene periods were predicted using both MIROC-ESM and CCSM4 models (Watanabe et al., 2011; Shields et al., 2012). Strongly correlated bioclimatic layers (*r* > 0.9) as determined using Pearson’s correlation analysis in R 3.6.2 (Appendix S3) (R Development Core Team, 2013) were excluded. Moreover, Maxent was independently performed among these species using area under the receiver operating characteristic curve (AUC) prediction model evaluation (DeLong, DeLong & Clarke-Pearson, 1988; Fawcett, 2006).

## Results

**Figure 1:**
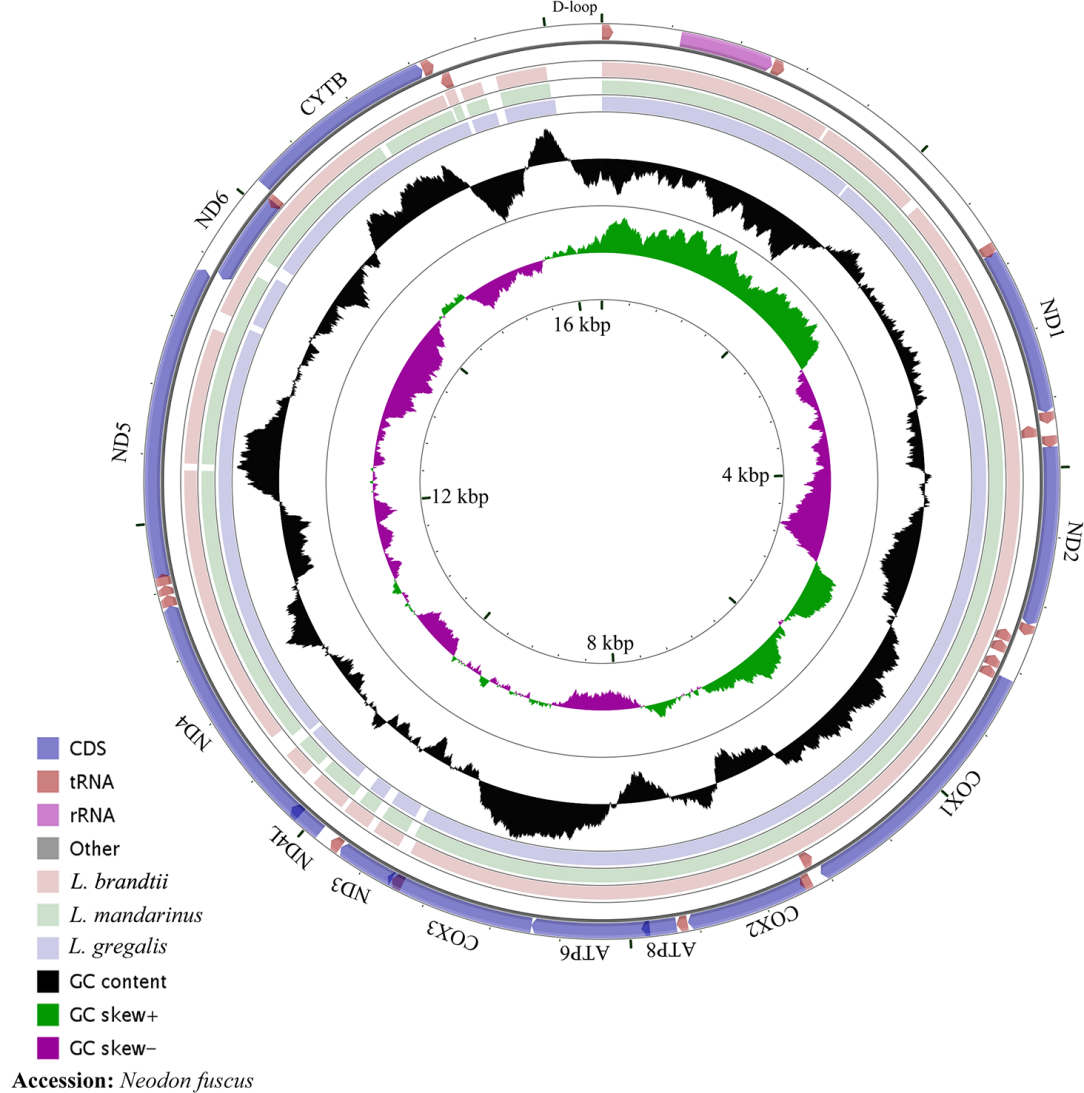
The complete mitochondrial genome map and GC skew of *Neodon fuscus*, *Lasiopodomys brandtii*, *L. mandarinus*, and *L. gregalis*.

The whole mitochondrial genome length of *L. mandarinus* was 16,562 bp, with the same sequences among repeated individuals. The mitochondrial genome length of *L. brandtii* was only 5 bp shorter than that of *L. mandarinus*, whereas that of *N. fuscus* was 220 bp shorter than that of *L. mandarinus* (Fig. 1). On the other hand, *L. mandarinus* was found to be 234 bp longer than the former sequenced mitogenomes (GenBank no. KF819832& JX014233). All sequences of the three species were longer than those of *L. gregalis*, a species previously in the genus *Microtus*, with sequence lengths of 16,292 bp (GenBank no. MN199169) and 16,294 bp (GenBank no. MN199170). All the three mitogenomes were assembled into a typical circular map with 13 PCGs, 22 tRNAs, 2 rRNAs (rrn12 and rrn16), and a D-loop region (Fig. 1, Table 1). Five types of start codons—ATA, ATC, ATG, ATT, and GTG—were identified among the PCGs, whereas three types of stop codons were identified for these species.

**Table 1:** Characteristics of the mitochondrial genome of Neodon fuscus, *Lasiopodomys brandtii, L. mandarinus*, and *L. gregalis*.

Genes	Position (bp)				Strat/stop codon			
	*L. brabdtii*	*L. mandarinus*	*L. gregalis*	*Neodon fuscus*	*L. brabdtii*	*L. mandarinus*	*L. gregalis*	*Neodon fuscus*
trnF-GAA	1-66	1-66	1-66	1-66				
rrn12	69–1017	69–1018	69–1017	69–1015				
trnV-UAC	1019–1087	1019–1088	1018–1087	1016–1086				
rrn16	1088–2641	1089–2652	1088–2649	1087–2648				
trnL-UAA	2650–2724	2655–2729	2651–2725	2650–2724				
ND1	2710–3681	2715–3686	2726–3680	2725–3679	GTG/TAG	GTG/TAG	GTG/TAG	GTG/TAG
trnI-GAU	3680–3748	3685–3752	3681–3748	3680–3747				
trnQ-UUG	3746–3817	3750–3821	3746–3817	3745–3816				
trnM-CAU	3820–3888	3823–3891	3820–3888	3818–3886				
ND2	3889–4923	3865–4926	3889–4923	3887–4921	ATC/TAA	ATC/TAA	ATT/TAA	ATC/TAA
trnW-UCA	4925–4991	4928–4994	4925–4991	4923–4989				
trnA-UGC	4993–5061	4996–5064	4993–5061	4991–5059				
trnN-GUU	5064–5133	5067–5136	5064–5133	5062–5131				
trnC-GCA	5168–5235	5171–5237	5167–5234	5163–5230				
trnY-GUA	5236–5302	5238–5303	5235–5301	5231–5297				
COX1	5268–6848	5296–6849	5303–6847	5299–6843	ATG/TAA	ATG/TAA	ATG/TAA	ATG/TAA
trnS-UGA	6846–6914	6847–6915	6845–6913	6841–6909				
trnD-GUC	6918–6985	6919–6986	6918–6985	6913–6980				
COX2	6978–7670	6979–7671	6987–7670	6982–7665	ATG/TAA	ATG/TAA	ATA/TAG	ATG/TAA
trnK-UUU	7674–7737	7675–7738	7674–7738	7669–7732				
ATP8	7738–7941	7739–7942	7739–7942	7733–7936	ATG/TAA	ATG/TAA	ATG/TAA	ATG/TAA
ATP6	7899–8579	7900–8580	7900–8580	7894–8574	ATG/TAA	ATG/TAA	ATG/TAA	ATG/TAA
COX3	8474–9412	8508–9413	8580–9363	8574–9357	ATG/TAG	ATG/TAG	ATG/TAG	ATG/TAG
trnG-UCC	9363–9430	9364–9431	9364–9431	9358–9426				
ND3	9431–9778	9432–9779	9432–9779	9427–9774	ATT/TAA	ATT/TAA	ATT/TAA	GTG/TAA
trnR-UCG	9780–9846	9781–9847	9781–9847	9776–9842				
ND4L	9849–10145	9851–10147	9850–10146	9844–10140	ATG/TAA	ATG/TAA	ATG/TAA	ATG/TAA
ND4	9962–11521	10141–11523	10140–11517	10134–11511	ATG/TTA	ATG/TTA	ATG/TTA	ATG/TTA
trnH-GUG	11517–11583	11519–11584	11518–11585	11512–11579				
trnS-UCU	11584–11642	11585–11643	11586–11644	11580–11638				
trnL-UAG	11642–11711	11643–11712	11644–11713	11638–11707				
ND5	11691–13523	11692–13524	11714–13525	11708–13519	ATT/TAA	ATT/TAA	ATA/TAA	ATA/TAA
ND6	13520–14104	13521–14147	13522–14046	13516–14040	ATG/TTA	ATG/TTA	ATG/TTA	ATG/TTA
trnE-UUC	14042–14110	14046–14114	14047–14115	14041–14109				
Cytb	14113–15258	14117–15262	14121–15263	14115–15257	ATG/TAA	ATG/TAA	ATG/TAA	ATG/TAA
trnT-UGU	15260–15326	15265–15331	15265–15331	15260–15327				
trnP-UGG	15566–15633	15522–15589	15332–15399	15328–15395				

The nucleotide composition of *L. brandtii*, *L. mandarinus*, and *N. fuscus* was biased for A+T by 59.5%, 59.5%, and 58.4%, respectively. All these mitogenomes showed a positive AT skew of 0.08 for *L. brandtii*, 0.09 for *L. mandarinus*, and 0.09 for *N. fuscus*. However, these species showed a negative GC skew ranging from −0.30 for *L. brandtii* to −0.34 for *L. mandarinus* (Fig. 1, Table 2). *L. gregalis* showed higher AT skew (0.10) and GC skew (−0.30) compared with the other three species. Among the 13 PCGs in these 4 species, nucleotide composition ranged from −0.69 in *ATP8* to −0.16 in *ND4L* for *L. mandarinus*, with a GC skew ranging from −0.14 in *ND4L* for *L. brandtii* to 0.33 in *ND6* for *L. mandarinus*. Similarly, all 13 PCGs exhibited a negative GC skew; however, *COX1*, *ND4L* in all species, *COX3* in *L. brandtii* and *L. mandarinus*, and *ND3* in *N. fuscus* showed a negative AT skew and *ND3* in *L. brandtii* and *L. mandarinus* had an AT skew of 0 (Table 2).

**Table 2:** Nucleotide composition data for the PCGs and whole mitochondrial genomes of *Neodon fuscus, Lasiopodomys brandtii, L. mandarinus*, and *L. gregalis*.

Species	contents	T	C	A	G	GC skew	AT skew
*L. brabdtii*	*whole*	27.4	26.4	32.1	14.1	−0.30	0.08
	*ATP6*	28.3	29.8	31	10.9	−0.46	0.05
	*ATP8*	26	27	37.7	9.3	−0.49	0.18
	*COX1*	29.5	25.5	27.1	18	−0.17	−0.04
	*COX2*	26.3	27.7	31.6	14.4	−0.32	0.09
	*COX3*	29.3	26.8	28.6	15.4	−0.27	−0.01
	*cytB*	27	29.1	30.5	13.4	−0.37	0.06
	*ND1*	28.5	28.9	30.7	11.9	−0.42	0.04
	*ND2*	26.7	31	33.9	8.4	−0.57	0.12
	*ND3*	30.7	26.1	30.5	12.6	−0.35	0.00
	*ND4*	27.8	28.3	31	12.9	−0.37	0.05
	*ND4L*	31.3	30.3	23.6	14.8	−0.34	−0.14
	*ND5*	28	27.8	32.4	11.8	−0.40	0.07
	*ND6*	20.6	30.9	38.6	9.9	−0.51	0.30
*L. gregalis*	*whole*	26.5	27.2	32.1	14.2	−0.31	0.10
	*ATP6*	17.6	30.5	29.8	12	−0.44	0.26
	*ATP8*	24.5	29.4	38.7	7.4	−0.60	0.22
	*COX1*	28.7	26.5	26.9	18	−0.19	−0.03
	*COX2*	28.3	26.1	30	15.6	−0.25	0.03
	*COX3*	27.6	28.4	29.1	14.9	−0.31	0.03
	*cytB*	26.5	29.7	30.3	13.5	−0.38	0.07
	*ND1*	25.9	31.5	30	12.6	−0.43	0.07
	*ND2*	26.1	29.7	35	9.3	−0.52	0.15
	*ND3*	27.3	29.9	30.7	12.1	−0.42	0.06
	*ND4*	27.1	29.4	31.9	11.4	−0.44	0.08
	*ND4L*	30.6	31.3	26.6	11.4	−0.47	−0.07
	*ND5*	25.9	30.4	31.5	12.3	−0.42	0.10
	*ND6*	22.4	29.4	39.6	8.7	−0.54	0.28
*L. mandarinus*	*whole*	27.1	27.1	32.4	13.4	−0.34	0.09
	*ATP6*	29.8	29.2	30.7	10.3	−0.48	0.01
	*ATP8*	27.9	28.9	37.7	5.4	−0.69	0.15
	*COX1*	28.7	26.5	27.7	17.1	−0.22	−0.02
	*COX2*	27	27.1	32.5	13.4	−0.34	0.09
	*COX3*	29	28.4	28	14.6	−0.32	−0.02
	*cytB*	26.5	30.7	30.6	12.1	−0.43	0.07
	*ND1*	28.4	29.1	30.2	12.2	−0.41	0.03
	*ND2*	26.9	30.9	33.9	8.3	−0.58	0.12
	*ND3*	32.2	24.1	32.2	11.5	−0.35	0.00
	*ND4*	28	28.5	32.3	11.2	−0.44	0.07
	*ND4L*	32	29.3	26.6	21.1	−0.16	−0.09
	*ND5*	27	28.9	33	11.2	−0.44	0.10
	*ND6*	20	30.7	40.1	9.2	−0.54	0.33
*Neodon fuscus*	*whole*	26.5	27.2	31.9	14.4	−0.31	0.09
	*ATP6*	27.6	31.3	28.8	12.3	−0.44	0.02
	*ATP8*	27	27	37.7	8.3	−0.53	0.17
	*COX1*	29	26.4	26.6	18	−0.19	−0.04
	*COX2*	26.8	26.8	31.5	14.9	−0.29	0.08
	*COX3*	27.1	29.5	28	15.4	−0.31	0.02
	*cytB*	25.8	31.3	28.8	14	−0.38	0.05
	*ND1*	25.9	30.7	31	12.4	−0.42	0.09
	*ND2*	25.7	30.7	35	8.6	−0.56	0.15
	*ND3*	29.6	28.2	28.2	14.1	−0.33	−0.02
	*ND4*	27	29.1	31	12.9	−0.39	0.07
	*ND4L*	29.2	30.2	26.8	13.8	−0.37	−0.04
	*ND5*	26.2	29.8	31.5	12.5	−0.41	0.09
	*ND6*	21.8	28.8	39.9	9.4	−0.51	0.29

**Figure 2:**
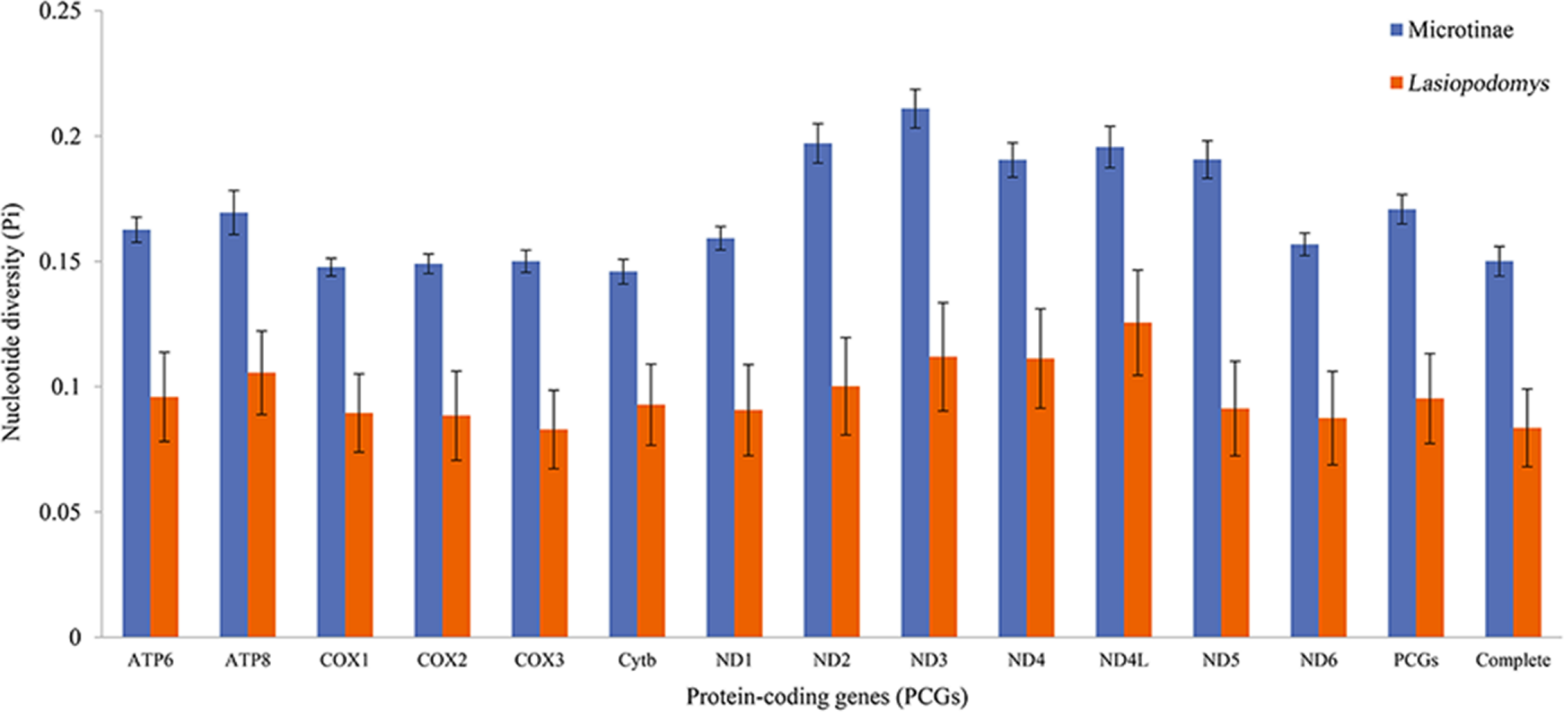
Nucleotide diversity of each protein-coding gene (PCG), concatenate PCG, and whole mitochondrial genomes of Microtinae (blue) and *Lasiopodomys* (orange).

The nucleotide diversity among the published Arvicolinae mitogenome sequences and our study species was 0.1429 ±  0.0001, whereas the nucleotide diversity of the mitogenomes of *Lasiopodomys* was 0.0836 ± 0.0155 (Fig. 2). The total nucleotide diversity in all 13 PCGs of Arvicolinae and the genus *Lasiopodomys* was 0.1603 ± 0.0027 and 0.0953 ±  0.0180, respectively (Fig. 2). In Arvicolinae, nucleotide diversity ranged from 0.1378 ±  0.0049 in *Cytb* to 0.1977 ± 0.0077 in *ND3*, whereas for *Lasiopodomys*, it ranged from 0.0829 ±  0.0157 in *COX3* to 0.1256 ± 0.021 in *ND4L*.

**Figure 3:**
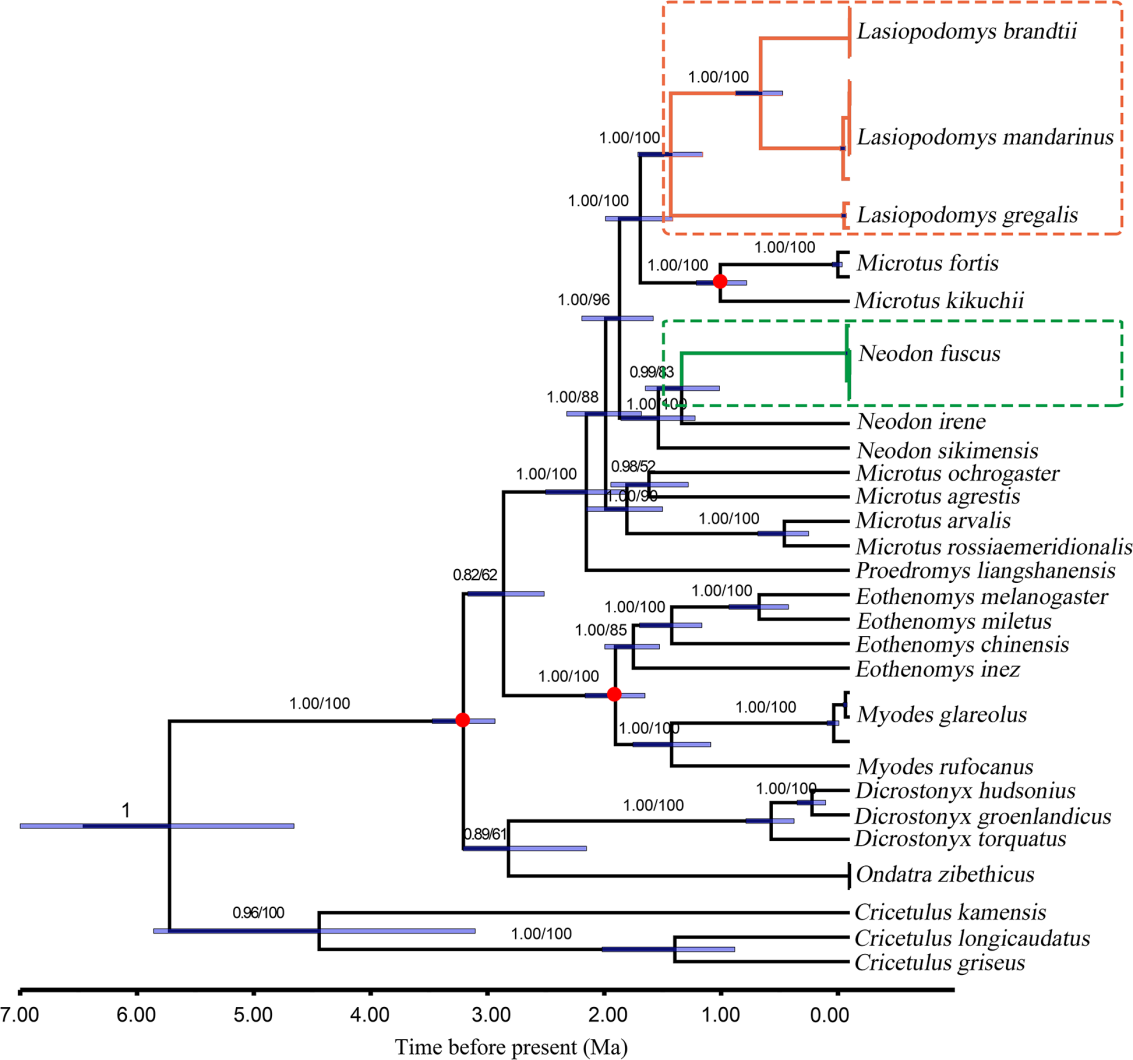
Divergence time for *Lasiopodomys* with whole mitochondrial genomes. The numbers on each node are posterior probabilities and bootstrap values. Blue bars show 95% highest posterior density intervals of node heights. Three red circles were fossil time. The genus of *Cricetulus* was used as an outgroup.

The results of the ML and Bayesian approaches were applied to the datasets of the whole mitogenomes, and the 13 PCG matrices inferred the same topology of the phylogenetic tree structure (Fig. 3). Our results supported that *Lasiopodomys*, *Microtus*, and *Neodon* have close relationships with the basal group of *Proedromys* Thomas 1911. Furthermore, the phylogenetic tree suggested that *L. brandtii*, *L. mandarinus*, and *L. gregalis* formed the genus of *Lasiopodomys*, whereas *N. fuscus* showed a close relationship with *N. irene*, belonging to the genus *Neodon*. *Microtus* was subdivided into two groups: one containing *M. fortis* and *M. kikuchii*, which were strongly supported as the sister group to *Lasiopodomys*, and the other was the basal group of the above species.

In the branch models, the one-ratio model was determined as superior to the *ω* = 1 model (*df* = 1, *p* <  0.01), suggesting that all the PCGs in the mitogenomes of *Lasiopodomys* undergo purifying selection (Table 3). In the branch-site model, only the ATP6 gene was present in some positive selection sites (60I 0.987, *p* < 0.01) in *Lasiopodomys* (Table 3). Moreover, positive selection sites were predicted in *Cox1*, *Cox3*, *Cytb*, *ND2*, *ND3*, and *ND5*. However, the LRTs were not significant.

The species divergence time among the *Lasiopodomys* species and related genera was calculated using the uncorrelated relaxed molecular clock model, which was calibrated with three prior divergence times of Arvicolinae (Fig. 3). The results suggested that the origin of *Lasiopodomys* was no earlier than the early Pleistocene epoch (∼0.781–2.58 Ma), with a possible most common ancestor of *Lasiopodomys* at ∼1.79 Ma (95% HPD values: ∼1.52–2.09 Ma). The split between *L. brandtii* and *L. mandarinus* was dated to the early Pleistocene period at ∼0.76 Ma (95% HPD values: ∼0.58–0.98 Ma), whereas the separation of both from *L. gregalis* was dated to the early Pleistocene epoch at 1.53 Ma (95% HPD values: ∼1.26–1.81 Ma). The estimated divergence event of *N. fuscus* and *N. irene* was found to be during the early Pleistocene epoch at 1.44 Ma (95% HPD Interval: ∼1.12–1.75 Ma).

**Figure 4:**
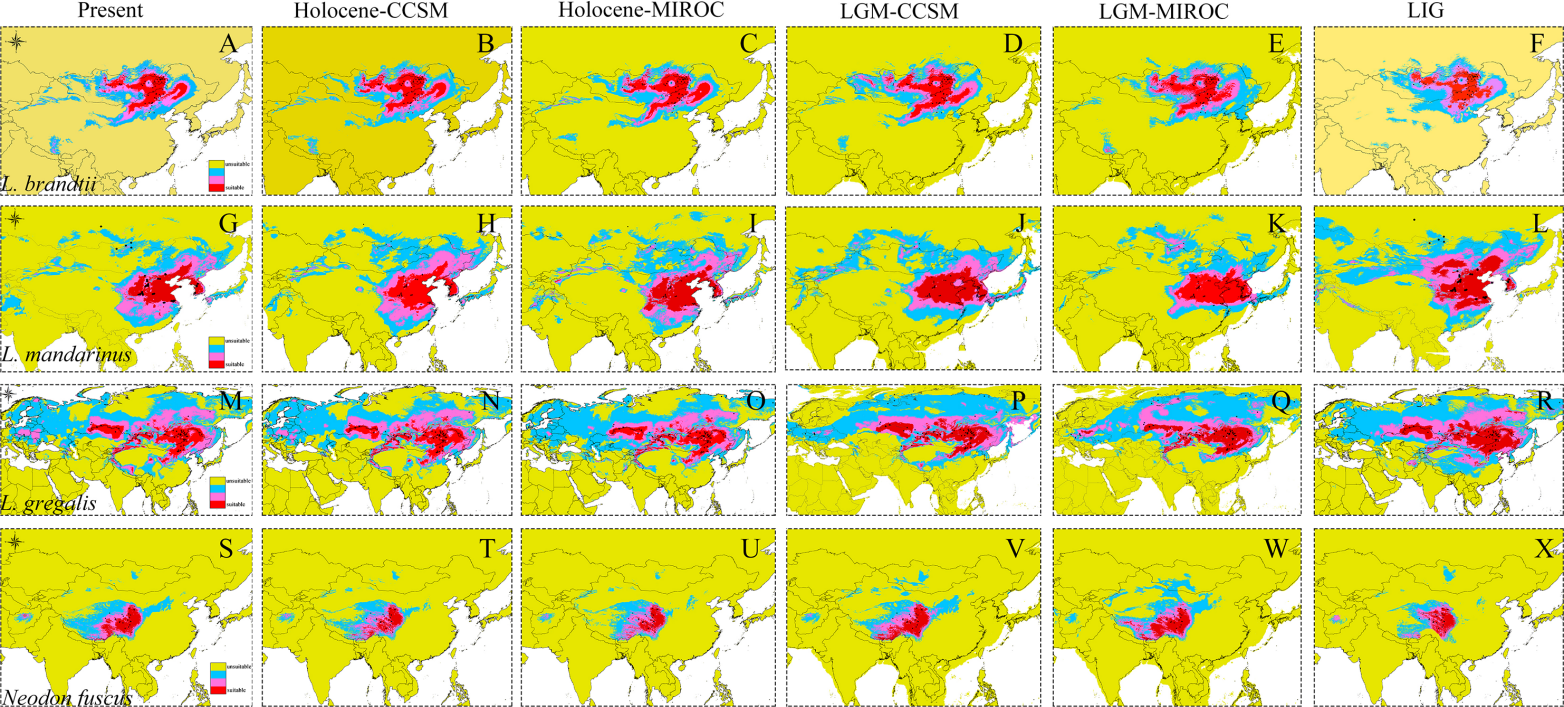
Ecological niche modeling of *Lasiopodomys* and *Neodon*. *Lasiopodomys brandtii* (A–F), *L. mandarinus* (G–L), *L. gregalis* (M–R), and *Neodon fuscus* (S–X) under the current climate and three periods in the past: the mid-Holocene, the Last Glacial Maximum (LGM), and the Last Interglacial Maxima (LIG).

The high AUC values determined via ecological niche modeling (ENM) indicated the good performance of the model predictions of this study (Appendix S4). During the periods from the LIG to present, all species of *Lasiopodomys* showed no evident loss of a suitable habitat. A western expansion of *L. brandtii* has been predicted in Northeast China, Inner Mongolia, and South Siberia, whereas a weak fragment was predicted for *L. gregalis* among the Eurasia regions (Fig. 4). Moreover, suitable areas were predicted in highly suitable habitat regions during the LGM in these species. More northern suitable areas were predicted during the LIG, and a northern expansion was predicted during the transition from the Holocene period to the present (Fig. 4). In addition, highly suitable habitats were observed for *N. fuscus* in the Hengduan Mountains during all periods, whereas more eastern distributions were predicted during the LGM (Fig. 4).

**Table 3:** Likelihood ratio tests of branch models and branch-site models examining the proteincoding genes of the genus *Lasiopodomys*.

Gene	Model		lnL	Models compared	Parameter Estimates	LRT ( *P*-value)
ATP6	Branch-model	A:One-ratio	−6321.793406		*ω*= 0.02625	*p* < 0.01
		B:Omega = 1	−7802.578602	B vs A	*ω*=1	
	Branch-site model	Null	−6298.662308	null vs A	7 A 0.578	P<0.01
		Model A	−6295.340396		60 I 0.987*	
ATP8	Branch-model	A:One-ratio	−2156.664937		*ω*=0.16120	*p* < 0.01
		B:Omega = 1	−2304.668614	B vs A	*ω*=1	
	Branch-site model	Null	−2092.811906			1
		Model A	−2092.811906	null vs A	NA	
Cox1	Branch-model	A:One-ratio	−12806.04872	B vs A	*ω*=0.00534	*p* < 0.01
		B:Omega = 1	−17316.74494		*ω*=1	
	Branch-site model	Null	−12712.24034	null vs A	57 I 0.779	0.077
		Model A	−12710.67689		487 T 0.965*	
Cox2	Branch-model	A:One-ratio	−5779.531238		*ω*=0.01386	*p* < 0.01
		B:Omega = 1	−7512.338542	B vs A	*ω*=1	
	Branch-site model	Null	−5711.411586			1
		Model A	−5711.411586	null vs A	NA	
Cox3	Branch-model	A:One-ratio	−6864.103188		*ω*=0.01989	*p* < 0.01
		B:Omega = 1	−8741.926003	B vs A	*ω*=1	
	Branch-site model	Null	−6757.123306			
		Model A	−6757.116417	null vs A	50 N 0.642	0.9065
					62 V 0.517	
					203 F 0.593	
Cytb	Branch-model	A:One-ratio	−10097.89327		*ω*=0.02761	
		B:Omega = 1	−12504.74705	B vs A	*ω*=1	*p* < 0.01
	Branch-site model	Null	−10010.08824			
		Model A	−10009.07827	null vs A	4 M 0.976*	0.1552
					7 K 0.892	
					116 I 0.567	
					242 V 0.522	
					315 I 0.516	
ND1	Branch-model	A:One-ratio	−9200.160474		*ω*=0.02426	
		B:Omega = 1	−11391.13236	B vs A	*ω*=1	*p* < 0.01
	Branch-site model	Null	−9015.80101			
		Model A	−9015.745075	null vs A	NA	0.738
ND2	Branch-model	A:One-ratio	−11468.97809		*ω*=0.06165	
		B:Omega = 1	−13190.51757	B vs A	*ω*=1	*p* < 0.01
	Branch-site model	Null	−11268.21175			
		Model A	−11268.21175	null vs A	11 F 0.747	1
					14 F 0.816	
					31 I 0.845	
					95 T 0.837	
					122 I 0.856	
					207 I 0.845	
					220 H 0.867	
					228 K 0.847	
					235 N 0.860	
					241 L 0.858	
ND3	Branch-model	A:One-ratio	−4086.367921		*ω*=0.06686	
		B:Omega = 1	−4686.550566	B vs A	*ω*=1	*p* < 0.01
	Branch-site model	Null	−3969.046821			
		Model A	−3969.013478	null vs A	6 A 0.811	0.7962
					14 S 0.790	
					20 V 0.861	
					108 Q 0.849	
ND4	Branch-model	A:One-ratio	−15050.32692		*ω*=0.04173	
		B:Omega = 1	−17886.34941	B vs A	*ω*=1	*p* < 0.01
	Branch-site model	Null	−14856.89331			
		Model A	−14856.89325	null vs A	NA	0.992
ND4L	Branch-model	A:One-ratio	−3210.084127		*ω*=0.05007	
		B:Omega = 1	−3775.223753	B vs A	*ω*=1	*p* < 0.01
	Branch-site model	Null	−3151.8857			
		Model A	−3151.8857	null vs A	NA	1
ND5	Branch-model	A:One-ratio	−19894.46685		*ω*=0.04666	
		B:Omega = 1	−23436.47839	B vs A	*ω*=1	*p* < 0.01
	Branch-site model	Null	−19737.31375			
		Model A	−19737.31225	null vs A	194 E 0.512	0.9563
					575 K 0.969*	
ND6	Branch-model	A:One-ratio	−5081.893461		*ω*=0.06927	
		B:Omega = 1	−5814.462234	B vs A	*ω*=1	*p* < 0.01
	Branch-site model	Null	−4971.821282			
		Model A	−4971.821283	null vs A	NA	1

## Discussion

### Structural features of the whole mitochondrial genome of *Lasiopodomys*

Among the nine complete mitochondrial sequences, all the species showed same sequences in the three repeated individuals, thereby supporting the accuracy and low intraspecific variation of our studies (Brown & Simpson, 1981). Although *N. fuscus* showed similar characteristics to previously sequenced mitogenomes (GenBank no. MG833880), *L. mandarinus* exhibited a longer sequence than that previously reported (Cong et al., 2016; Li, Lu & Wang, 2016a; Li et al., 2016b; Li et al., 2019). This difference may be due to nucleotide errors, particularly in tandem repeats, caused by different sequencing technologies: Sanger sequencing versus high-throughput sequencing (Pfeiffer et al., 2018). All these differences occurred in the intergenic region, with little impact on subsequent analysis. Therefore, we reserved both types of sequence data in the subsequent analysis.

All the PCGs of these species, similar to the other Arvicolinae mitogenomes, had an incomplete stop codon that was automatically filled during the transcription process in the mitogenomes of animals, with no effect on translation (Ojala, Montoya & Attardi, 1981). Similar to previous studies, the nucleotide diversity of all the PCGs in both *Lasiopodomys* and Arvicolinae typically showed the highest divergence in the NADH dehydrogenase complex and the lowest divergence in the cytochrome c oxidase subunit complex and cytochrome B gene (Huang et al., 2019; Ramos et al., 2018). The nucleotide sequence diversity of the NADH dehydrogenase gene groups may be affected by variations in the historical environment (Ramos et al., 2018; Mueller, 2006). Similar to previously published mitogenomes, the AT skew of *Lasiopodomys* and *N. fuscus* was consistent with that of vertebrates (Zhang, Cheng & Ge, 2019; Martin, 1995), further indicating evolutionary pressure related to the mechanism of DNA replication (Charneski et al., 2011; Dai & Holland, 2019).

### Phylogenetic relationships of *Lasiopodomys*

Our molecular phylogenetic analysis results were highly consistent those of previous studies. In our study, the subfamily Arvicolinae was supported as a monophyletic group based on the molecular data of *Cytb*, *COX1*, *GHR*, *CLOCK*, and *BMAL1* (Abramson et al., 2009b; Buzan et al., 2008; Liu et al., 2017; Martin et al., 2000; Sun et al., 2018). Our results suggest that *N.* (*Lasiopodomys*) *fuscus* within the genus *Neodon* forms a sister relationship with *N. irene*, consistent with the results reported by Chen et al. (2012) and Li et al. (2019). The stable clustering of *L. brandtii*, *L. mandarinus*, and *L. gregalis* into one group confirms the systematic positions of *Lasiopodomys*. This topology was consistent with that of other phylogenetic studies based on nuclear genes (Sun et al., 2018), mitochondrial DNA (Abramson et al., 2009a; Liu et al., 2012b; Martínková & Moravec, 2012; Petrova et al., 2016), and whole genomes (Li, Lu & Wang, 2016a; Li et al., 2019; Tian et al., 2020). However, it contradicts with the systematic position based on the morphological characteristics of these species (Allen, 1940; Corbet, 1978; Wilson & Reeder , 2005). Further, *L. brandtii* and *L. mandarinus* have consistently presented as a sister group in molecular phylogenetic studies, with seldom distinguished morphological characteristics but different aboveground and underground habitats, suggesting a mechanism of environmental adaptation during rapid speciation (Alexeeva, Erbajeva & Khenzykhenova, 2015; Dong et al., 2018; Li et al., 2017). Other species of *Microtus* and *Neodon* were not found in the monophyletic group (Liu et al., 2012a); *M. kikuchii* and *M. fortis* were grouped as sister lineages within the *Lasiopodomys* clades and were considered belonging to the subgenus *Alexandromys* based on phylogenetic research (Mezhzherin, Zykov & Morozov-Leonov, 1993), allozymes, and *Cytb* (Bannikova et al., 2010). All these genera form a “*Microtus s.* l.,” which could be the “core Arvicolinae” (Baca et al., 2019).

### Evolution and demographic history of *Lasiopodomys*

When inferring the divergence time of *Lasiopodomys* and related genera, both the Yule process and birth–death process speciation models were required with multiple fossil calibration nodes employed in phylogenetic analysis to develop more robust estimates (Drummond & Rambaut, 2007; Humphreys et al., 2016). Based on complete genomes and PCG phylogenetic trees, both models presented similar estimates of a relatively recent origin and divergence time for *Microtus s.* l. during the early Pleistocene epoch. The oldest reported fossil of *Microtus s.* l. was during the early Pleistocene epoch (Chaline et al., 1999). An arid and cold environment raised species dispersal and speciation in response to Pleistocene climatic fluctuations (Vasconcellos et al., 2019). Our study supported the first appearance of *Lasiopodomys* in the late early Pleistocene epoch from the Transbaikal area (Alexeeva, Erbajeva & Khenzykhenova, 2015; Li et al., 2017) at ∼1.52–2.09 Ma (Petrova et al., 2016) but later than that estimated by chromosomes at 3 Ma (Gladkikh et al., 2016). At ∼1.28–1.81 Ma, the morphological characters of *L. gregalis* proposed the earliest clades of modern *Lasiopodomys*, as indicated by molecular data and fossils (Abramson et al., 2009a; Chaline et al., 1999; Petrova et al., 2016). Thereafter, the clades separated into *L. brandtii* and *L. mandarinus* at ∼0.58–0.98 Ma in our study, which is similar to inferences from *Cytb* and D-loop sequences (Li et al., 2017; Petrova et al., 2015) but less similar to the inferences from molecular cytogenetic analyses at ∼1.8 Ma (Gladkikh et al., 2016).

ENM indicated a considerably wider distribution area of *Lasiopodomys* in the past than in the present, which conforms to the fossils from the Pleistocene period (Alexeeva, Erbajeva & Khenzykhenova, 2015). During the early Pleistocene period, continuous cooling formed an arid climate in the high latitudes of the Northern Hemisphere (Guo et al., 2008). Climatic changes seldom shifted the suitable habitat of *Lasiopodomys* during the LIG and LGM periods. It is possible to infer that migration events occurred during the extremely cold and dry conditions, with a trend of continuous distribution farther to the northeast during the Pleistocene period until the Holocene period (Alexeeva, Erbajeva & Khenzykhenova, 2015; Prost et al., 2013). The appearance of *N. fuscus*, which is adapted to plateau climates, was later than the Qinghai-Tibet Plateau uplift (Wang et al., 2008), with no significant distributed shifts. All ancient species of *Lasiopodomys* may have been distributed as per their current distribution areas with a radiation evolution (Abramson et al., 2009b; Bannikova et al., 2010) before the interglacial and glacial periods based on ENM and fossil reports (Alexeeva, Erbajeva & Khenzykhenova, 2015; Petrova et al., 2015). Considering the lower sensitivity to climatic changes and adaptation to habitat areas, the *Lasiopodomys* species could colonize in north regions; moreover, the evolution of characteristics, such as teeth and densely furred plantar surfaces, further enabled their survival in cooler, drier conditions.

Despite precipitation and temperature fluctuations, a decline in atmospheric O_2_also occurred during the past 0.8 Ma (Stolper et al., 2016). Environmental stress caused a major driving on evolutionary process (Parsons, 2005). In the species of rodents, limited oxygen availability resulted in evolutionary adaptation and appearance of various strategies (Pamenter et al., 2020), such as different colonial habitats of life—subterranean (*L. mandarinus*) and plateau (*L. fuscus*); these strategies formed unique physiological and molecular adaptations to hypoxia (Jiang et al., 2020; Dong, Wang & Jiang, 2020). Our study supports a history of rapid population expansion under positive selection via mitogenome sequences such as the ATP6 gene, which uses oxygen to create adenosine triphosphate. However, further research using integrated phylogeographic analyses of the genus *Lasiopodomys* (Li et al., 2017; Petrova et al., 2015) is warranted to determine the adaptation of *L. brandtii* and *L. mandarinus* to factors including precipitation, temperature, and chronic hypoxia.

##  Supplemental Information

10.7717/peerj.10850/supp-1 Supplemental Information 1 List of species used in this study and their accession numbers in GenBank

10.7717/peerj.10850/supp-2 Supplemental Information 2 All presence records among *N. fuscus*, *L. brandtii*, *L. mandarinus*, and *L. gregalis*

10.7717/peerj.10850/supp-3 Supplemental Information 3 Correlated bioclimatic values using Pearson’s correlation analysis

10.7717/peerj.10850/supp-4 Supplemental Information 4 Receiver operating characteristic curve (ROC) values for *N. fuscus*, *L. brandtii*, *L. mandarinus*, and *L. gregalis* under ecological niche models

10.7717/peerj.10850/supp-5 Supplemental Information 5 Original data on the mitochondrial genome of nine samples

## Additional Information and Declarations

### Data Availability

The following information was supplied regarding data availability: Sequences are available at GenBank: MT614214 to MT614219. Sequences are also available in the Supplementary Files.
